# Buffalo Academy of Medicine

**Published:** 1903-07

**Authors:** N. L. Burnham


### SOCIETY PROCEEDINGS.

### Buffalo Academy of Medicine.

*Section on Medicine, Tuesday Evening, February 10, 1903.*

Reported by N. L. BURNHAM, M. D., Secretary.

The medical section of the Buffalo Academy of Medicine held its meeting on Tuesday evening, February 10, 1903.

The second paper of the evening was read by Dr. Irving M. Snow, entitled

### INJURIES AND INFECTIONS OF NEWLY-BORN CHILDREN.

The paper was divided into two parts:

I. *Infant Mortality in the First Month of Life.* — The first four weeks may be regarded as the most dangerous period of human life. Nearly one-tenth of the race succumb in the first month of existence. Unrecognised intracranial hemorrhage, atelectasis, or pneumonia are common causes of death in infants. The susceptibility to disease lessens with each month of life. Victims of congenital weakness, malformations, injuries of parturition, die first, and the surviving continually gain resistance to disease.

MORTALITY BY MONTHS OF THE FIRST YEAR OF LIFE—NORWEGIAN STATISTICS.

| | | | |
|---|---|---|---|
| 1st month | 3.41 per cent. | 7th month | .52 per cent. |
| 2d month | .99 " " | 8th month | .50 " " |
| 3d month | .76 " " | 9th month | .47 " " |
| 4th month | .67 " " | 10th month | .45 " " |
| 5th month | .62 " " | 11th month | .41 " " |
| 6th month | .56 " " | 12th month | .40 " " |

Total, 9.76 per cent. in first year.

The same excessive death-rate exists in Buffalo. During the year ending December 1, 1902, 9.3 per cent. of all children born in Buffalo died in the first four weeks, and the death-rate in the first month forms 6.4 per cent. of the total mortality of these children; 38.5 per cent. dies in the first 24 hours; 30 per cent. in second to seventh day of life; 16.5 per cent. dies second week; 7.3 per cent. in third week; 7.6 per cent. in the fourth week.

In Norway, where the infant mortality is the lowest in the world, we find that among 1,000,000 births in the rural districts, from 1876 to 1898, the death-rate in the first month was 3.38 per cent. of all children born. In Prague the mortality in the first month is 14.2 per cent.; in Prague hospital it is 3.3 per cent., showing conclusively that the life of the newly-born child is most favorably influenced by good midwifery and nursing. Statistics show that more children die with us from accidents of parturition, and fewer from infections, than in Europe. The mortality in early life is due to (1) immaturity; (2) malformations incompatible with life; (3) asphyxia and atalectasis; (4) injuries of parturition; (5) various infections. Cerebral hemorrhage is the most frequent lesion found in infants stillborn or dying soon after birth. Application of forceps, nipping of a cerebral sinus in forcible molding of the head, foot and breech presentations, softness of the skull and relaxation of sutures, are the most frequent agents in producing hemorrhage.

Dr. Snow reported a series of cases illustrating the clinical history of intracranial hemorrhage. The usual symptoms are cyanosis, rapid and irregular breathing, temperature, opisthotonic rigidity, quivering, automatic movements, convulsions, baby apathetic in the intervals of relaxation. The child may die in a few days or live to be a paralytic or an epileptic. The majority die before the fourth day. Treatment of intracranial hemorrhage is almost hopeless. Even if the convulsions are relieved, the residual disabilities are not lessened. Asphyxia and atalectasis are fertile causes of bronchopneumonia and formed 3.6 per cent. of causes of death in infants during the first month of life.

Sepsis is a frequent cause of death in the newly-born. The avenues of infection are the eye, ear, nose, mouth, skin, umbilicus, digestive and respiratory tracts. The healthy placenta is a perfect filter for germs. The amniotic fluid polluted by the examining finger, or other sources, is the greatest source of danger after the bag of waters is broken. The germ is generally the colon bacillus. Inflammatory conditions in the respiratory or gastrointestinal tracts may result. A majority of the infants dying in the first month have a bronchopneumonia. It is diagnosticated principally by objective signs, fever, cyanosis, and rapid breathing. There are no characteristic physical signs in the chest.

II. *Gastroenteric infections at birth.*—Gastroenteric infections at birth are frequent and important. Congenitally imperfect digestion exists in some babies. The digestive ferments are weak. The congested gastroenteric tract with a hypersecretion of mucus hinders the complete assimilation of food, favors autointoxication or infection from without. The congenitally sterile gastroenteric tract is normally invaded by bacteria in fourteen hours after birth. Dr. Snow believes severe gastroenteric infection more frequent and fatal than inflammation of the umbilical region. In Buffalo, in the year ending December 1, 1902, there were among infants under one month 30 deaths from gastroenteric infection, and 40 from marasus and inanition—16 per cent. of the total mortality. Dr. Snow then reported 10 cases of gastroenteric infection. None of the mothers showed the slightest sign of illness, and he arrived at the conclusion that the infants were infected after birth. Nine of the mothers attempted to nurse their babies. In one the history of feeding was not noted. Five of the children died and five recovered. The vehicle of contagion is most probably either the milk of the mother or amniotic fluid contaminated by the colon bacillus. All but one occurred in private practice and among clean, intelligent people.

Treatment—Gastric and intestinal lavage, calomel, stimulation, hydrotherapy and a wet nurse.

DISCUSSION.

Dr. DeLancey Rochester opened the discussion, saying that he blamed the large infant mortality in a large number of cases to poor care after birth, and not to antenatal injury or disease. More care should be exercised by physicians in signing infants' death certificates.

Dr. L. Schroeter called attention to the injudicious use of instruments as a cause of cerebral hemorrahge in infants. He did not believe the statistics were reliable. Many stillborn children among the Poles and Italians, on account of religious belief, are baptized in utero on the hand, the foot or any presenting part of the fetus, or even the cord, by midwives, and these cases swell the number of infants classed as having died within a few days after birth. Syphilis of the newborn is not frequently stated on death certificates, even when it is known to exist.

Dr. F. Thoma referred to nephrolithiasis as a probable cause of death in some infants who die within 40 to 72 hours following suppression of urine.

Dr. A. J. Colton referred to hemorrhage of the bowels, strangulated hernia, hematuria, probably due to uric acid occurring in his practice and causing death in infants.

Dr. J. S. Jones thought we should consider heredity as an etiological factor in infant mortality. An improperly nourished pregnant woman cannot be expected to be the mother of a well nourished child capable of resisting disease.

Dr. B. G. Long said the statistics reflect rather discreditably upon Buffalo, and thought they were unreliable. Norwegians have not large families. We have a mixed population.

Dr. Rochester, replying to a question from Dr. Snow, said that the use of chloroform upon the mother during labor did not act as a cause of death of the child. He always uses chloroform unless otherwise requested, and does not use forceps in 5 per cent. of his cases.

Dr. Snow closed the discussion. Amniotic fluid polluted by the examining finger may be the cause of death in the newly-born. Aspiration pneumonia is sometimes a cause. The meconium is sterile for 14 to 15 hours after birth. The Norwegians are a very prolific race. Rachitis is extremely common in Prague, where the mortality in the first month corresponds with Norway. Cerebral hemorrhage is a frequent cause of death in infants. Uric acid infarcts do not cause death.

Replying to Dr. Schroeter, he said he did not think congenital syphilis common in Buffalo, as it is exceedingly rare in the newlyborn at the Erie County hospital. He does not think stillborn children are reported as of full term. He thinks the statistics are correct. The point in regard to the injudicious use of forceps is well taken.

Fellows present, 18. Meeting adjourned 10.30 p.m.

---

### *Stated Meeting, May 26, 1903.*

Reported by F. W. McGUIRE, M. D., Secretary.

A stated meeting of the Academy of Medicine was held Tuesday evening, May 26, 1903. The president, Dr. Herman E. Hayd, called the meeting to order at 9 p.m. The minutes of the last stated meeting, held March 31, 1903, and the minutes of the special meeting, held May 20, 1903, were read and approved.

On motion the meeting was adjourned for ten minutes to allow the section on obstetrics and gynecology to elect officers for the ensuing year.

The program of the evening was furnished by the section on obstetrics and gynecology, which was as follows:

SHOULD THE UTERUS BE REMOVED WHEN OPERATING FOR DOUBLE PUS TUBES? Matthew D. Mann, M. D.

PROPHYLAXIS IN OBSTETRICS. Ludwig Schroeter, M. D.

These papers were discussed by P. W. Van Peyma, W. Hartwig, M. C. Breuer, S. Goldberg, E. E. Blaauw, H. B. Singer, F. W. McGuire, Herman E. Hayd, and Matthew D. Mann.

Attendance, 20. Adjourned at 10.30 p.m.

